# Puerperal Infection*Read before the Clinical Society of St. James Dispensary, December 4, 1897.

**Published:** 1898-02

**Authors:** St. Joseph B. Graham

**Affiliations:** Savannah, Ga.


### PUERPERAL INFECTION.*

BY ST. JOSEPH B. GRAHAM, M.D.,

SAVANNAH, GA.

If this paper were exhaustively written it would consume more time than can just at present be given in writing it, and more than you can, perhaps, afford in listening to it when read. Therefore, an attempt only is made to touch upon what we consider the most important under the scope of the paper.

The causes of puerperal infection are various and can be classified as follows: Infection from streptococcus pyogenes, usual cause; staphylococcus pyogenes aureus and albus; Klebs-Lœffler bacillus of diphtheria; bacillus coli communis; gonococcus of Neisser of gonorrhea, and perhaps the bacillus of malignant edema (vibrion septique Pasteur).

The manner or way of introduction of the micro-organisms is twofold: either from the patients themselves or their dressings, or, the most usual, from the hands, instruments or dressings of the physician or nurse.

It seems quite out of place to discuss at length all of these well-known sources.

The leading infection from patients themselves might be further elaborated by mention of the rôle played by the birth of dead and macerated feti, retained placenta, in part or whole, accessory growths

* Read before the Clinical Society of St. James Dispensary, December 4, 1897.

of the placenta, and spurious placenta, all of which favor infection by furnishing a favorable soil for the growth and multiplication of micro-organisms.

It is plain that in order to have puerperal infection certain of these lower organisms must be present, and they gain admission either through a solution of continuity, small or large, or through the puerperal endometrium. The virulence of these organisms varies much, due to differences in temperature, source and growth, and the infection varies with this condition, as well as with the number of microbes present, and the individual susceptibility of the patient.

The pathology of puerperal infection depends upon the species of organism producing the infection. In ordinary cases the infection is due to the streptococcus pyogenes, and the most easily noted change is that of the blood even before and especially after death. It is thick and black, is acid in reaction, and decomposes quickly. Streptococci are found in it at times in large numbers, as well as in other organs and tissues of the body. The leucocytes and red corpuscles are disintegrated, partly by the organisms themselves, as well as by the toxin elaborated by them. In consequence of this change in the blood and in the blood-vessels, numerous hemorrhagic foci take place in the internal organs. In the intestines we may find enteritis or ileo-colitis; in the heart pericarditis, endocarditis; in the kidneys an acute inflammatory catarrhal condition; in the uterus metritis, in the veins phlebitis. Changes in the internal organs are not however constant. When the infection is a mixed one, we have a pyemic condition combined with it where metastatic abscesses are present through the different tissues and organs of the body.

### TREATMENT.

Of first and greatest importance is the preventive treatment—its causes usually being under our control. We must begin prophylaxis when we can see any indication for the same at any time before the period of labor. Any condition or disease, local or constitutional, that lowers the vitality of the patient or furnishes a nidus for bacteria certainly predisposes to infection, and we may include auto-intoxication in the list. These should be met by proper therapeutic, surgical and hygienic measures, and if possible relieved or cured.

Any abnormal secretion from the vagina calls for proper treatment as soon as recognized.

We often find a leucorrhea due solely to systemic disturbance, which resists, of course, local treatment, but is readily cured when the real cause is recognized. In chronic malarial toxemia the author has often seen this condition, which readily yields to proper doses of iron, quinine and arsenic.

Of course, if gonorrhea (recognized by gonococci) is present, treatment will at once be resorted to. In health the vagina secretes a mucus and cultivates saprophytic bacilli, which render it immune to the invasion and harmful influences of pathogenic bacteria; therefore, in health preliminary antiseptic douches are not only uncalled for but are harmful, in that they upset or destroy the provisions of nature.

As an antiseptic for the hands the writer prefers a two or three per cent. solution of formalin (a forty per cent. solution of formaldehyde) gained by the incomplete oxidation of methyl alcohol dissolved in water. The nails are first well cleaned (to prevent what a witty friend a few days ago remarked, making cultures from the finger-nails before visiting the case to decide what kind of serum to use). No matter how clean they may appear, a new or sterilized nail brush and sand soap is used for the fingers, hands and arms, afterwards washed in alcohol and thoroughly washed and soaked in the formalin solution. It is needless to say that nothing unsterilized should be touched after the cleansing. A sterilized gown should be worn by physician and nurse. Everything should be gotten in readiness beforehand. The writer begs to show you here a little sterilizer for instruments when needed, of his own evolution, which is compact, cheap, easy to transport and fulfils the purpose for which it is intended. With the small alcohol stoves the water will be boiling in five minutes; a two per cent. soda solution may be used and formalin may be added if desired. Make as few vaginal examinations as possible and wash the hands in formalin solution after each examination, and when a lubricant is needed use sterilized cottonseed-oil or vaseline.

The writer had the good fortune to serve a few years in the frontier service as physician, being at a post among the Sioux Indians, and there noted the extreme rarity of puerperal infection among them, despite the filth and unhygienic condition of the surroundings of puerperal women. They never permit the introduction of the finger for examination, or the hand for aid, except in the most extreme cases, and these cases were of such a nature that death frequently came before delivery. In the few cases of infection the wauseca pejutawicasa (the white medicine man) had been in attendance.

The patient's external genitals should be thoroughly scrubbed. Most of our patients do not neglect their body bath. Where they do it is well to remind them of its usefulness, aside from common cleanliness. The woman in and after labor should be treated with the same regard for asepticism and surgical cleanliness as we would use in the most extensive operation wound of our own making, where the surface was previously free from infection.

Unless the hands have been in the uterus, which is to be avoided if possible, by Créde's method of expression, or there is some indication for its use, the writer would not resort to intrauterine douches. When labor and the soft parts are normal, and the rules of asepticism have been rigidly adhered to, vaginal douches can do no good.

The treatment of infection should be local and constitutional, surgical and medical, influenced of course by the variety of microorganisms producing the condition. The parts should be examined as far as possible by the eye to determine the focus and in a measure the classification of infection, and upon the information gained the treatment will depend. For local disinfection the writer prefers a one to four per cent. solution of formalin to any other antiseptic known. Where the point of infection is in the uterus it must be decided whether it is sapremic or septicemic. Clearing and cleaning out is indicated; the surgically clean finger, dull curette or sharp curette may be used (each has its advocates and disadvantages), followed by prolonged douching with or without germicidal agents. Here again the writer prefers formalin solution. It is non-toxic, but very slightly irritating in proper strength, and as far as germicidal properties are concerned, heads the list.

If remains of necrosed tissue are not present, curetting will accomplish nothing.

If the case is seen early, before septic absorption and migration of bacteria have taken place to any great extent, the writer prefers to use a blunt dilator and irrigator combined, which I here beg to present to you. This, after sterilization, is introduced and opened, the irrigating portion having been filled with liquid to prevent the introduction of air. It is attached to an irrigating bottle or funnel and a solution of formalin allowed to flow in, while the dilator is moved in every possible direction. This may be maintained as long as desired, being careful not to have the solution too strong and the hydrostatic pressure too great by too high an elevation of the container. The temperature afterwards should be the guide as to the length of time and value of the washing out. Formaldehyde or iodoform gauze may be used to induce drainage and act as germicidal agents, or, as iodoform does, to prevent the formation of toxins. An original plan has presented itself to the writer, but which an opportunity has not offered for use. It is the conveying of formaldehyde gas, combined with vapor of alcohol, into the uterine cavity through a suitable tube uterine applicator. Theoretically, it should prove of great value; practically, we do not know what it will do. At any rate it is non-toxic to the patient. Steam at 100 to 115 C. has been used in this manner with reported good results.

The constitutional treatment is modified in a measure by the kind of infection present.

If diphtheritic, due to Klebs-Lœffler bacilli, anti-diphtheria serum should be used. If, as more often happens, the pseudo-membranous angina and the poisoning is due to streptococcus pyogenes, anti-streptococcus serum is indicated, that of Marmorek probably being the most noted, but many serums made in our own country are as good.

Ten cases are reported of streptococcus phlegmon where the swelling of lymphangitis and lymphadenitis quickly disappeared after the use of serum.* The serum treatment, however, seems not

*Dieudenna. Schutzimpfung and serum therapie. Leipsic, 1895.

to have yet reached that high grade of healing power to which it is thought and hoped it will later rise.

The indications are to sustain by proper remedial agents and stimulants, judiciously employed, the patient's vitality until the *vis medicatrix naturæ* sufficiently asserts itself. The writer believes in pushing alcoholic stimulants.

With the report of one case I beg to close my brief remarks. Patient, age thirty-nine, delivered at full term of twins, dead three or four weeks, *in utero* and attached to one placenta, which came away entire; no laceration of uterus or soft parts. Next day lochia, apparently normal. On fourth day, at 6 A. M., severe chill, followed by temperature of 105, pulse 148, stupor. Used dilator as above described and irrigated with formalin solution for one hour. A few small shreds came away, but nothing else. Temperature shrank to 103 in the afternoon, when irrigation was again resorted to. Temperature at 9 P. M. 102. Irrigation again next day; temperature 102, in afternoon 101; irrigation. Temperature at 9 P. M. 100. Morning irrigation; temperature 100¼; afternoon temperature 100⅘; irrigation; night temperature normal. Irrigation next morning. Patient bright, appetite returning, and went on to recovery.

Blood examination showed no plasmodii, but excess of leucocytes. Calomel in small doses and quinine were given internally.

